# Left ventricular noncompaction cardiomyopathy and short QT syndrome due to primary carnitine deficiency

**DOI:** 10.1111/anec.13077

**Published:** 2023-09-01

**Authors:** Oliver P. Hanington, Catherine Armstrong, Germaine Pierre, Graham Stuart, Jules C. Hancox

**Affiliations:** ^1^ Cardiovascular Research Laboratories, School of Physiology, Pharmacology and Neuroscience University of Bristol Bristol UK; ^2^ Bristol Heart Institute Bristol UK; ^3^ Bristol Royal Hospital for Sick Children Bristol UK

**Keywords:** cardiomyopathy, carnitine deficiency, short QT syndrome

## Abstract

We report the case of a 13‐year‐old female patient presenting with presyncope and palpitations. Her electrocardiogram revealed an abbreviation of the rate‐corrected QT interval with imaging showing significant left ventricular dysfunction. Carnitine levels were measured as part of her diagnostic workup, discovering a rare, reversible cause of short QT syndrome (SQTS) and associated cardiomyopathy—primary carnitine deficiency (PCD) caused by a homozygous mutation in the *SLC22A5* gene, leading to an in‐frame deletion mutation (NP_003051.1:p.Phe23del) affecting the organic cation transporter 2 (OCTN2) protein. Following the treatment with oral carnitine supplementation, her QT interval returned to within the normal range with significant improvement in left ventricular function.

## Graphical abstract

A 13‐year‐old patient with palpitations and syncope was diagnosed with a rare, reversible cause of short QT syndrome (SQTS) and cardiomyopathy—primary carnitine deficiency (PCD) caused by a mutation in the *SLC22A5* gene. Treatment with carnitine supplementation corrected her QT interval to within normal range, with significant improvement in ventricular function.

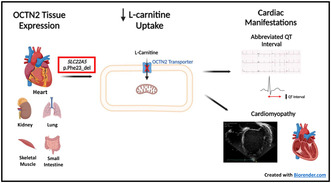

## 1 INTRODUCTION

Long‐chain fatty acids (LCFAs) are important energy substrates in muscular tissue, but they are unable to diffuse freely across the inner mitochondrial membrane. L‐carnitine is a key metabolic cofactor, driving carnitine palmitoyltransferase I, a rate‐limiting step in mitochondrial uptake and oxidation of LCFAs (Fu et al., 2013; Hancox, 2018). Organic cation transporter 2 (OCTN2), encoded by the *SLC22A5* gene (OMIM 603377) (Fu et al., 2013), is responsible for the active uptake of L‐carnitine into several tissues, in particular the heart, skeletal muscle, and kidney. Mutations in the *SLC22A5* gene cause primary carnitine deficiency (PCD; OMIM 212140), a rare autosomal recessive condition. Primary carnitine deficiency is characterized by deficiency in intracellular carnitine, low plasma carnitine, and high urinary carnitine due to renal wasting (Fu et al., 2013; Hancox, 2018). Primary carnitine deficiency commonly manifests in cardiac dysfunction, with the development of a progressive cardiomyopathy that can lead to end‐stage heart failure unless the condition is diagnosed and treated with carnitine supplementation (Fu et al., 2013; Shibbani et al., 2014).

In 2016, Roussel and colleagues reported an association between PCD and abbreviated rate‐corrected QT (QTc) intervals, with tall T waves (Roussel et al., 2016) reminiscent of those seen in patients with ion channel mutations leading to short QT syndrome (SQTS) (Hancox et al., 2018). An independent report of a young girl with progressive cardiomyopathy and left ventricular dilatation has also indicated an association between PCD and QT_c_ abbreviation with tall T waves (Perin et al., 2018). This patient was heterozygous for an *SLC22A5* deletion mutant, which sufficed to lower blood carnitine and cause PCD. Both her cardiomyopathy and QT_c_ abbreviation responded to dietary carnitine supplementation. Here, we report a new case of concurrent QT_c_ interval abbreviation and cardiomyopathy due to PCD, responsive to dietary carnitine supplementation.

## 2 CASE REPORT

**FIGURE 1:**
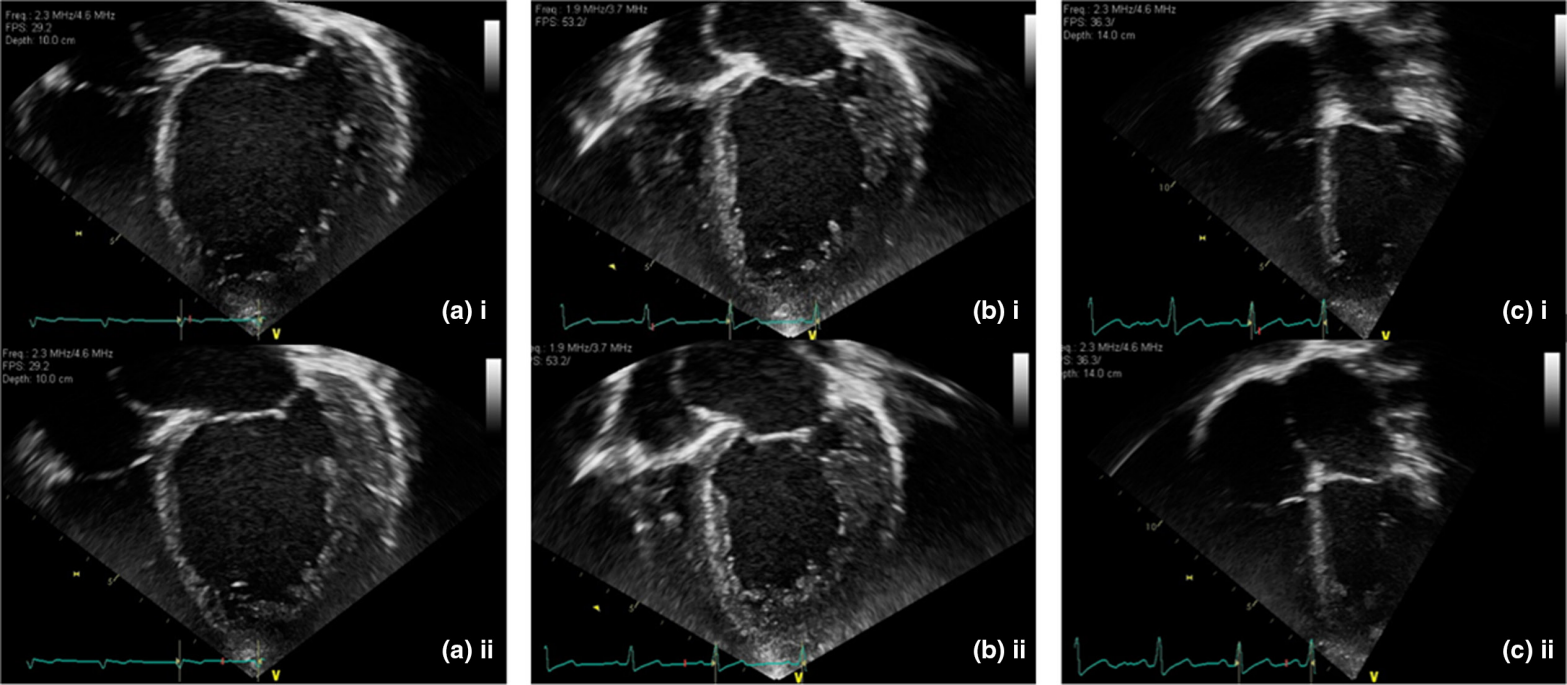
Apical four‐chamber transthoracic echocardiogram. (a) ECHO at time of diagnosis, (b) after 8 weeks of carnitine supplementation, (c) after 2 years of carnitine supplementation. (i) end‐diastole, (ii) end‐systole.

**FIGURE 2:**
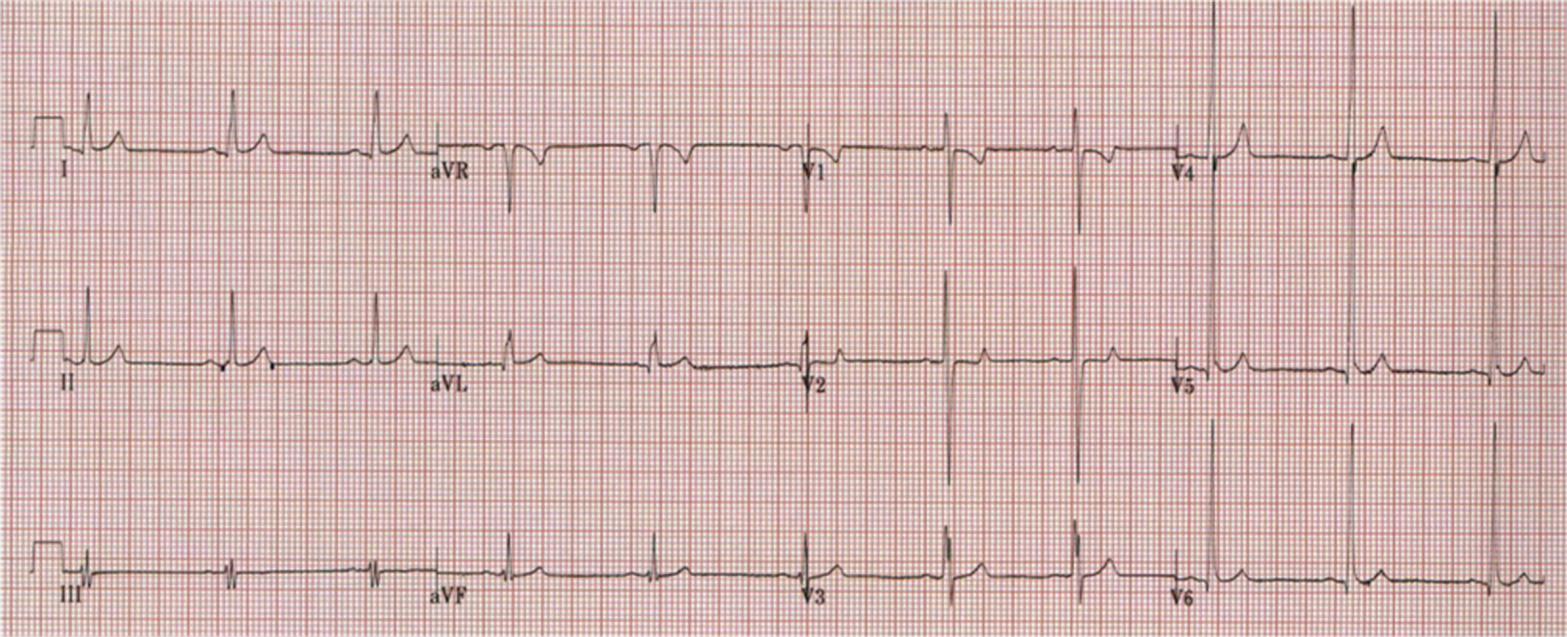
12‐lead electrocardiogram at time of diagnosis. Sinus bradycardia with a short QT interval (QT 280 ms; QT_c_ 280 ms) and peaked (6–10 mm) T waves with a J‐point to T‐peak interval of 160 ms.

The patient was a 13‐year‐old female refugee born to nonconsanguineous parents originating from Iraq. She presented with a history of presyncope and palpitations, having previously been diagnosed with an unspecified heart problem aged 9 years. On arrival in the UK, she was being treated with multiple heart failure medications, including carvedilol, digoxin, furosemide, and captopril. On examination, no dysmorphic features were present, with cardiac auscultation revealing a soft systolic heart murmur. Transthoracic echocardiography revealed significant left ventricular (LV) dilatation with severe LV dysfunction (ejection fraction [EF] 35%; mitral systolic plane excursion [MAPSE] 5 mm) and features of LV noncompaction (Figure 1ai–ii). There was a family history of sudden cardiac death, with her brother dying suddenly aged 3 years. There was no other history of cardiac disease in the family. A 12‐lead electrocardiogram (ECG) demonstrated sinus bradycardia (heart rate 60) and a shortened QT interval (QT and QT_c_ of 280 ms) with peaked T waves, suggestive of SQTS (Figure 2). This represented 5 points on the Gollob, short QT diagnostic criteria, score (Gollob et al., 2011).

Paired plasma and urine carnitine analysis showed negligible free plasma carnitine (2 and 116 μmol/L, respectively), with a tubular reabsorption of free carnitine of 22% (cf >90% in unaffected individuals). These features strongly suggested a diagnosis of PCD.

Genetic analysis was conducted using next‐generation sequencing targeting 153 genes associated with arrhythmia and cardiomyopathy, using the Agilent Focussed exome target enrichment kit. This revealed a homozygous deletion (HGVS description: NM_003060.3:c.67_69del; location: Chr5 [GRCh37]: g.131705731_131705733del) in the *SLC22A5* gene, resulting in an in‐frame deletion p.(Phe23del) in the OCTN2 protein. Together with the plasma carnitine and tubular reabsorption of free carnitine levels, this confirmed a diagnosis of PCD. Genetic testing of the three surviving siblings was requested to be performed by the adult metabolic services, but unfortunately the results were not available.

**FIGURE 3:**
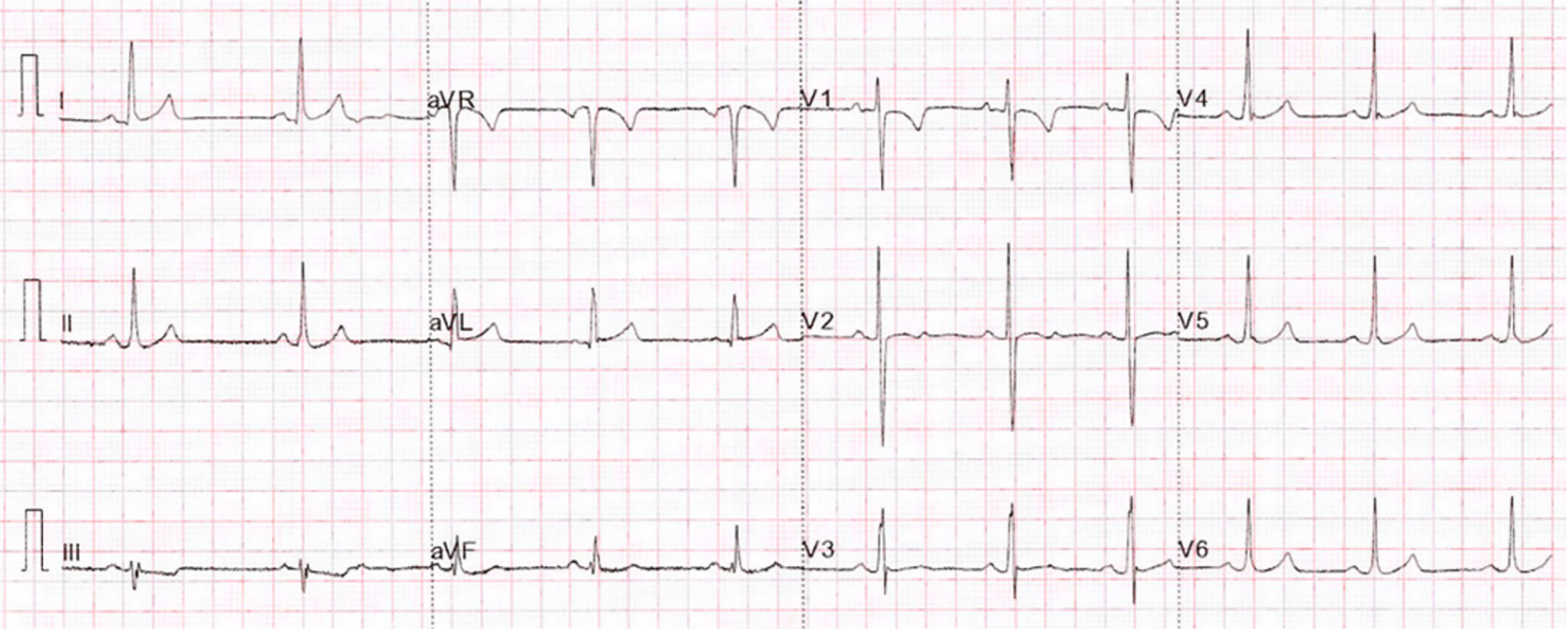
12‐lead electrocardiogram after 4 years of carnitine supplementation. Significant lengthening in the QTc interval is present (417 ms). Precordial T‐wave inversion present at 4‐month review has resolved.

The patient was treated with long‐term 1000 mg TDS carnitine supplementation. Electrocardiogram recording at 4 months showed her QT_c_ interval had returned to within the age‐specific normal range (QTc = 402 ms). While there was a general reduction in T‐wave amplitude, T‐wave inversion was present throughout the precordial leads. She was fitted with a Reveal‐Linq™ device, which identified no arrhythmias at 12‐month follow‐up. An echocardiogram after 2 years of carnitine supplementation showed a clear regression of cardiomyopathy (Figure 1ci–ii) with LV function having significantly improved (MAPSE = 9 mm; LV EF = 56%). A further ECG at 4 years post diagnosis and treatment (Figure 3) showed her QT_c_ interval remained within the normal range (QTc = 417 ms), with correction of the inverted precordial T waves.

## 3 DISCUSSION

Primary carnitine deficiency is a rare condition, with one study reporting an incidence of between 1 in 40,000 and 1 in 120,000 (Magoulas & El‐Hattab, 2012); its prevalence is highest in the Faroe Islands (1 in 297) (Rasmussen et al., 2014). The most frequent pathological variant in the *SLC22A5* gene is the missense mutation p.(Pro46Ser), with an allele frequency of 4.73e^−4^. In contrast, the p.(Phe23del) deletion mutation that is the subject of the current case report has an allele frequency of 1.97e^−5^, with no homozygote cases reported in the gnomAD database (Chen et al., 2022). To our knowledge, this is the first report to demonstrate carnitine‐sensitive QT_c_ interval shortening with the p.(Phe23del) OCTN2 mutation. In the study by Roussel and colleagues associating PCD with QT_c_ interval shortening, one patient was found with a premature stop codon mutation p.(Trp62*) on one allele and a p.(Arg471Cys) mutation on the other. His mother had the same p.(Arg471Cys) mutation and a complete deletion of exon 2 of *SLC22A5* on the second allele (Roussel et al., 2016). In the second study by Perin et al. (2018), reporting concurrent QT_c_ shortening and cardiomyopathy, the patient was heterozygous for a p.(Arg289*) truncation mutant (Perin et al., 2018). Recently, a p.(Phe17Leu) mutation was found on molecular autopsy of a young woman who died suddenly. This was also present in her brother who had been diagnosed with SQTS (Gelinas et al., 2019).

A heterozygous OCTN2 mutation p.(Phe23del) has been observed previously in a female child of East Indian / Irish origin with deletion of nucleotides 67–69 in one *SLC22A5* allele (NM_003060.3:c.67_69del) and a single nucleotide substitution (g.14344G>A) at a splice donor site at the 5′ end of intron 3 in the other allele (Lamhonwah et al., 2002). At age 3, she had features of heart failure with marked left ventricular dilatation and peaked T waves on the anterolateral precordial leads, with no QT_c_ data reported (Tein et al., 1990). She was initially treated with digoxin and diuretics and later, aged 4, was found to have low carnitine levels (total 19 μM; free 15 μM). Her cardiac dimensions and function improved on oral carnitine, with inversion of the formerly peaked T waves. By age 6.5 years, her digoxin and diuretics were discontinued. A separate study reported a Lebanese patient homozygous for p.(Phe23_del), who was aged 10, had an ejection fraction of 29% with cardiomyopathy and low normal carnitine levels (total 31.2 μM; free 24.7 μM; Shibbani et al., 2014). After 4 years of carnitine supplementation, his EF had improved to 59%. No ECG data were reported in that study. An adjacently positioned homozygous OCTN2 deletion mutation, p.(Phe22_del), has been reported in a symptomatic patient. However, accompanying QTc data were not provided (Rose et al., 2012).

A review of patients in the Faroe Islands, who have a particularly high prevalence of PCD (Rasmussen et al., 2020), suggests a strong association between *SLC22A5* mutations and the risk of sudden cardiac death (SCD). The risk of SCD was increased in female patients, with the etiology unclear. Kayıkçıoğlu et al. (2022) report the case of an 11‐year‐old patient with a p.(Arg169Gln) homozygous mutation, diagnosed during family screening. On stopping carnitine supplementation, he had episodes of syncope, which subsequently resolved on recommencing treatment.

Roussel et al. (2016) demonstrated a causal link between PCD and QT_c_ interval abbreviation through the generation of a mouse model of PCD using MET‐88 (also known as meldonium), which inhibits OCTN2 and L‐carnitine biosynthesis (Hancox, 2018; Roussel et al., 2016). Twenty‐eight days of treatment with MET‐88 led to reduced carnitine levels, cardiac hypertrophy, and QT interval abbreviation without changes in PR or QRS intervals. A proportion of treated mice also developed arrhythmias including sustained ventricular tachycardia (Roussel et al., 2016). However, cellular electrophysiology experiments to identify which ion channels are responsible for these changes have not yet been conducted. It is tempting to speculate that the intracellular LCFA accumulation arising from PCD modulates one or more of the ion conductances that regulate repolarization, to produce effects analogous to those of SQTS mutations (Hancox et al., 2018). Preclinical studies to better our understanding of the underlying electrophysiological mechanisms are warranted.

The present report demonstrates unequivocally that the homozygous p.(Phe23_del) mutation can lead to both cardiomyopathy and to a marked electrophysiological phenotype denoted by QT_c_ abbreviation and precordial T‐wave augmentation. The responsiveness of these changes in carnitine supplementation provides further evidence of a causal link between PCD and pathological QT_c_ shortening with this mutation.

Short QT syndrome is usually thought to occur without marked structural abnormalities (Hancox et al., 2018). However, the findings in our report, together with those of other recent studies (Gelinas et al., 2019; Perin et al., 2018; Roussel et al., 2016), strongly suggest that PCD should be considered as a potential cause of QT_c_ interval abbreviation/SQTS (or an “SQTS‐mimic”; Walsh et al., 2022/SQTS phenocopy), particularly in the setting of concurrent cardiomyopathy (Perin et al., 2018).

## 4 CONCLUSION

The p.(Phe23del) OCTN2 mutation led to reversible QTc abbreviation in this patient. This case further highlights that the diagnosis of atypical SQTS should take the link between PCD, cardiomyopathy, and QT_c_ abbreviation into consideration (Roussel et al., 2016; Shibbani et al., 2014).

## AUTHOR CONTRIBUTIONS

CA, GS, and GP involved in investigation and diagnosis. JCH gave advice on literature linking PCD to QT interval shortening. CA managed the patient. OPH involved in analysis. OPH and JCH wrote the initial draft. All authors involved in critical revision of the manuscript for intellectual content.

## CONFLICT OF INTEREST STATEMENT

The authors have no conflicts of interest to declare that are relevant to the content of this case report.

## CONSENT

Informed written consent was gained from the patient and patient's mother prior to submission of this case report.

## ETHICS STATEMENT

Ethical approval was not sought as this report contains one case report, and informed consent was obtained.

## Data Availability

The data that support the findings of this study are included in this published article.
